# Microneedles for Efficient and Precise Drug Delivery in Cancer Therapy

**DOI:** 10.3390/pharmaceutics15030744

**Published:** 2023-02-23

**Authors:** Keisheni Ganeson, Ain Hafizah Alias, Vikneswaran Murugaiyah, Al-Ashraf Abdullah Amirul, Seeram Ramakrishna, Sevakumaran Vigneswari

**Affiliations:** 1 Faculty of Science and Marine Environment, Universiti Malaysia Terengganu, Kuala Nerus 21030, Terengganu, Malaysia; 2 Centre for Drug Research, Universiti Sains Malaysia, Gelugor 11800, Penang, Malaysia; 3 Discipline of Pharmacology, School of Pharmaceutical Sciences, Universiti Sains Malaysia, Gelugor 11800, Penang, Malaysia; 4 School of Biological Sciences, Universiti Sains Malaysia, Gelugor 11800, Penang, Malaysia; 5 Malaysian Institute of Pharmaceuticals and Nutraceuticals, National Institutes of Biotechnology Malaysia, Gelugor 11700, Penang, Malaysia; 6 Centre for Chemical Biology, Universiti Sains Malaysia, Bayan Lepas 11800, Penang, Malaysia; 7 Center for Nanofibers and Nanotechnology, Department of Mechanical Engineering, National University of Singapore, Singapore 117581, Singapore; 8 Institute of Marine Biotechnology, Universiti Malaysia Terengganu, Kuala Nerus 21030, Terengganu, Malaysia

**Keywords:** cancer, microneedles, pain, transdermal, painless, gains

## Abstract

Cancer is the leading cause of death, acting as a global burden, severely impacting the patients’ quality of life and affecting the world economy despite the expansion of cumulative advances in oncology. The current conventional therapies for cancer which involve long treatment duration and systemic exposure of drugs leads to premature degradation of drugs, a massive amount of pain, side effects, as well as the recurrence of the condition. There is also an urgent demand for personalized and precision-based medicine, especially after the recent pandemic, to avoid future delays in diagnosis or treatments for cancer patients as they are very essential in reducing the global mortality rate. Recently, microneedles which consist of a patch with tiny, micron-sized needles attached to it have been quite a sensation as an emerging technology for transdermal application to diagnose or treat various illnesses. The application of microneedles in cancer therapies is also being extensively studied as they offer a myriad of benefits, especially since microneedle patches offer a better treatment approach through self administration, painless treatment, and being an economically and environmentally friendly approach in comparison with other conventional methods. The painless gains from microneedles significantly improves the survival rate of cancer patients. The emergence of versatile and innovative transdermal drug delivery systems presents a prime breakthrough opportunity for safer and more effective therapies, which could meet the demands of cancer diagnosis and treatment through different application scenarios. This review highlights the types of microneedles, fabrication methods and materials, along with the recent advances and opportunities. In addition, this review also addresses the challenges and limitations of microneedles in cancer therapy with solutions through current studies and future works to facilitate the clinical translation of microneedles in cancer therapies.

## 1. Introduction

Cancer has been the leading cause of death worldwide, accounting for approximately 10 million deaths in 2020, or nearly one in six deaths, with a total of 18.1 million cancer cases [1,2]. Cancer cases in Malaysia has also been increasing significantly with a total of 48,639 cases as of 2020, which accounts for almost 1 in 10 people in Malaysia [3]. The total cancer incidence is also expected to double by the year 2040. This is highly disappointing and surprising at the same time because most types of cancer can be effectively treated through current therapies, especially with all the technological advancements; nevertheless, cancer patients continue to experience an excruciating amount of pain and it remains to be a disease without a cure. Cancer also remains to be one of the most frightening and traumatizing experiences for anyone bearing this diagnosis, despite all the progress and advancements in prevention, early detection, and newer, more effective treatment modalities [4].

However, to this date, there has only been an increase in research studies emphasizing the biology of cancer and drug discoveries or novel formulations for treatments and understanding of their pharmacology. There are much fewer studies focused on understanding the effect of cancer diagnosis and treatments on the patients’ quality of life, as well as clinical studies on the scale of pain or pain management of current treatments [5]. The goal of future cancer treatments should take into serious consideration the patient’s comfort and function while avoiding unnecessary adverse effects and providing a pain-free treatment option. Quality of life and patient-centred therapy should be the main goals or priority of any medical sector as we march towards the fourth industrial revolution [6,7,8].

On top of that, there is an increasing global population and rising technology demand for a better healthcare system focusing on personalized and precision-based medicine [9,10,11]. Statistically, cancer incidence is expected to rise to 29.5 million, and mortality to 16.4 million is predicted over the next 40 years, which would make cancer a more serious epidemic [5,12,13]. This highlights the increasing importance of personalized medicine which is to provide the right treatment at the right time for every patient with improved diagnostic accuracy. Currently, cancer drug delivery is mostly delivered orally or via the parenteral route. Nevertheless, at advanced cancer stages, these routes may not be a feasible way to administer the drugs due to patients’ condition, and therefore researchers have explored novel ways to deliver drugs, one of which is via the microneedle.

**Figure 1:**
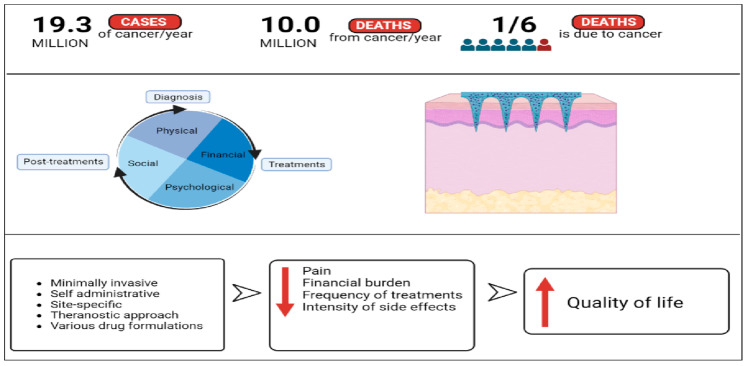
Schematic illustration of how the advantages of microneedles technology could reduce the common negative effects involved in cancer therapy and increase the quality of life of cancer patients (created using BioRender.com).

Microneedle technology is a unique modern transdermal therapy approach that consists of a patch with tiny, micron-sized needles attached to it which are designed to load vaccines, drug molecules, proteins, genes, antibodies, nanoparticles, and many more [14]. Microneedles can be used to successfully overcome the limitations associated with conventional treatment methods in cancer, while offering patients painless gains as they are advantageous from diagnosis to treatments and even theranostics. Figure 1 shows a schematic illustration of how cancer patients could gain without pain using microneedles.

Microneedles are also a self-administrative device that could transdermally uptake interstitial fluid, deliver drugs, vaccines, etc., in a minimally invasive and painless manner [15,16]. In addition, microneedles can also be utilized for a combinatory approach with two different therapies at the same time and can be fabricated and modified in any shape, size, and geometry or material depending on the use and release mechanism [16]. Since microneedles could be self-attached by patients, they could also offer a more precise and efficient personalized treatment approach for cancer patients. The ability of attaching microneedles directly or close to the targeted tumor area increase the precision of drug delivery to the tumor cells, thus increasing the efficiency of treatment. Microneedles have higher accuracy in tumor targeting and drug delivery in comparison to other conventional treatment approaches such as hypodermic injections or oral drug delivery.

This review offers an overview of microneedles as a painless technology and focuses on highlighting microneedles as the cutting-edge and idea-inspiring technology in biomedical engineering which could make cancer patients gain a quality of life without experiencing pain through a more targeted transdermal treatment approach. Undoubtedly, microneedles could revolutionize cancer treatments by enabling earlier cancer detection, enhancing treatment efficiency, as well as providing an improved quality of life. This review is believed to be valuable to the integrative notion contained in the hallmarks of cancer.

## 2. General Properties and Classification of Microneedles

Microneedles comprise a patch as the base support with micron-sized needles attached to it, and it has been getting a lot of attention in the research field for the past few decades [15]. The term microneedle was first introduced in 1998 but the concept of microneedle technologies was proposed as a transdermal drug delivery device by Pistor in the 1970s [17,18].

Typically, there are several hundred micrometer squares of microprojections on the microneedles resembling honeybee comb structures in the range of 500–900 μm length, 50–250 μm wide and 1–25 μm thickness [19,20,21]. Microneedle technologies have been produced by a variety of techniques, comprising different materials, shapes and characteristics for a broad range of applications such as cosmetics, drug delivery, diagnosis and many more. The type of microneedles, along with the methods of fabrication and materials, are interrelated to their application. Since their initial production up till now, a good number of studies for therapeutic, diagnostic and biomedical purposes have been conducted. Ever since the microneedle transdermal drug delivery system was introduced, many commercial microneedle technologies have come into the market; some common examples are summarized in Table 1.

### 2.1. Types of Microneedles

**Figure 2:**
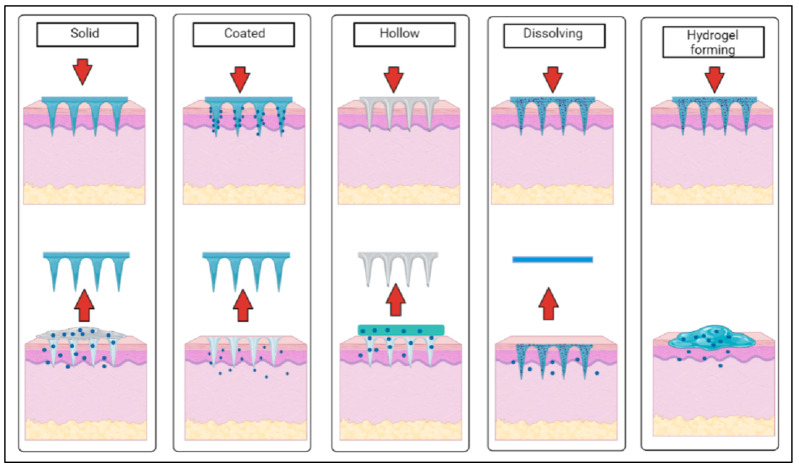
Schematic diagram of the mechanism of action of different types of microneedles after application on the skin (created using BioRender.com).

There are five main types of microneedles: solid, hollow, dissolving, coated and hydrogel-forming. Each type of microneedle array has a different mechanism of action after application on the skin [27], as shown in Figure 2 below.

Solid microneedles are mostly used in two parts with a poke and patch mechanism to deliver drugs into the body, where the first part involves the application of the microneedle array for the creation of microscopic pores as the conduit channels in the skin, and the second part is to apply drug formulation in various forms (e.g., topical cream, gel, solution and drug patch) after the removal of the microneedle arrays [21]. Coated microneedles have a coat and poke approach which tends to provide fast drug release through the fast dissolution of the coating layer of needles, whereas hollow microneedles have an empty core with a pore at the tip that functions as microfluidic channels that disrupt the layers of skin to transdermally deliver the drugs in a poke and flow manner or to extract biological fluids [21]. Dissolving and hydrogel-based microneedles are a recent class of microneedles categorized as polymeric microneedles where dissolving microneedles have a poke and release mechanism, while hydrogel-forming microneedles have a poke and swell approach when inserted within the skin [16].

The differences in types and mechanisms of actions also cause a variety of advantages and limitations, and thus they could serve different functions in cancer therapy. The type of microneedles and their advantages and limitations is summarised in Table 2 below.

Generally, solid and hollow microneedles are more preferred for diagnostics and theranostics approaches as they could draw out interstitial fluid from the body, whereas dissolving microneedles and hydrogel-forming microneedles are more preferred in treatments in terms of drug delivery, vaccine delivery and gene therapy as they could efficiently deliver the drugs.

### 2.2. Type of Materials and Fabrication Methods

The choice of materials in the fabrication of microneedles is strongly related to their applications as each material influences the microneedle features such as permeability, strength and flexibility, which affects the efficiency of therapeutic approaches [29]. The fabrication methods also depend on the type of materials used. Common materials used in manufacturing microneedles are metals, glass, ceramic, silicon, carbohydrate and polymer, which are all approved by U.S. Food and Drug Administration (FDA) [30]. The first microneedle arrays were fabricated from silicon, and since then, microneedle fabrication has steadily improved in recent years because of modern improvements and further advances in polymer chemistry and microengineering, encouraging innovative system designs in microelectronic technology and microfabrication [18].

There are a few ideal characteristics of these materials that should be considered to fabricate a microneedle that works systematically. The materials should have the capability to resist the insertion force during microneedle insertion and maintain the shape without breaking after insertion [20]. In addition, the materials should also be biocompatible, not induce toxicity [29], be compatible with the drug formulations, and economically as well as environmentally beneficial. Other than that, there are various fabrication methods for the manufacturing of microneedles. This includes wet etching, lithography, metal injection moulding, laser machining, micro-moulding, deep reactive ion etching, and thermal micro-moulding. Based on the types of microneedles, Table 3 summarises the materials and fabrication methods used, along with their advantages and limitations.

According to the comparisons in the table above, biopolymers seem to be more advantageous than the rest of the materials. They are highly preferred due to their biodegradability, biocompatibility, and low toxicity as compared to the other materials, and since they are mostly used in fabricating dissolving microneedle, it makes the dissolving microneedle arrays even more advantageous in cancer therapies.

In addition to the types, materials and methods used in the fabrication of microneedles, there are also other important features such as the uniformity of microneedle performance, structural strengths and geometric factors such as height, width, density, aspect ratio, platform flexibility and needle brittleness, tip thickness, uniform degradation, and signal transmissibility, which needs careful considerations [49,50]. This is because every feature of microneedles could affect their efficiency, especially if it is used for medical applications. This opens the possibilities for a wide variety of combinations in microneedles that has more enhanced properties for application in cancer therapies. However, chronic illnesses such as cancer should need more research on the optimisation of the features to effectively treat patients with minimal pain, as well as to avoid any side effects which might aggravate the tumor cells, keeping in mind the cost and sustainability.

## 3. Advantages and Limitations of Microneedle

Microneedles, the tiny but mighty delivery device which focuses on minimizing pain and maximizing gain are highly promising as they have countless advantages in the healthcare industry, but there are still limitations which should be addressed before commercializing it for use in cancer therapies.

### 3.1. Transdermal Route of Administration

The key concept of a microneedle-based therapeutic system involves the transdermal route of administration, which offers a precise and targeted site-specific drug delivery. The transdermal route of administration is when active ingredients are delivered across the skin for a systemic distribution, and despite being deeply studied over the past 50 years, it has once again become popular with the microneedle system. It is considered as one of the robust routes for drug delivery compared to the other routes of administration (e.g., oral, topical, parental, etc.), especially in cancer treatments as it has shown great promise for the site-specific attack on tumor cells and reducing systemic toxic effects [15].

The skin serves as a reservoir in transdermal drug delivery enabling the diffusion of the penetrated drugs to the deeper epidermis and dermis without drug accumulation in the dermal layer continuously over a longer period to achieve the controlled and sustained release of drug candidates that have short biological half-lives and require a high frequency of administration [49]. The distinctive physiological structure of the skin presents an excellent opportunity for this due to the wealth of blood and lymphatic vessels in the skin, which is well connected to the rest of the body [51]. Microneedle arrays are minimally invasive devices which pierce the stratum corneum layer, disrupt the skin barrier, and reach up to the epidermis layer and then enter the blood vessels to directly reach the target site of action, without reaching the pain nerves [51]. Transdermal treatment approaches with microneedles have more advantages due to their characteristics of avoiding first-pass metabolism and controlling the rate of drug input over a prolonged time, especially in chronic illnesses such as cancer.

Microneedles can be applied anywhere on the body similarly to a patch system, making the diagnosis and drug delivery in cancer treatments more efficient compared to hypodermic injections through the parental route. This is because the drugs administered under the skin will rapidly join the blood flow and cause the drug level at the targeted region to remain over hours or days, whereas the drugs administered through hypodermic needles undergo rapid degradation as they have to travel longer in the body system before reaching the targeted cancer cells, lowering the bioavailability of administered drugs/vaccines, which in turn increases the need for higher or frequent doses to get the intended effect. The site-specific targeting of localized tumor cells through microneedles is also beneficial for the delivery of cancer medications with a narrow therapeutic window or short half-lives [52]. However, the ability of microneedles to completely replace the current hypodermic needles, especially in future cancer care which includes diagnostics, treatments and pain management, is highly debatable as it depends on multiple factors.

The site-specific approach of microneedles does have certain limitations in their use in cancer therapy, especially in diagnosis. Allergic reactions or skin inflammation is usually caused by locally accumulated drugs, the reduced penetration of drugs, limited microneedle access to plasma tumor markers, microbial contamination, and local itching [19,53,54]. These could also be caused by the sensitivity or skin conditions of patients. Nevertheless, the reports on microbial contaminations after the use of microneedles are very limited and are often associated with defects in construction, the immunogenicity of components or the application of microneedles [49]. The pores created by microneedles are also very small as compared to that of a hypodermic needle, thus requiring lesser time to heal and showing lesser microbial penetration, and could be minimized with polymer- or hydrogel-based microneedles or by the addition of antimicrobial agents during the fabrication of microneedles [19]. It is also quite normal for the skin to experience slight swelling around the application site as the skin layer is disrupted while a foreign material is being inserted into the skin. Moreover, the skin’s elasticity and tension lead to indentation occurrence when there is an incomplete insertion of microneedles, which could eventually lead to limited drug delivery efficiency and wastage of valuable medicines [55,56]. Movements, stretching and bending of skin later could also cause the incomplete insertion of microneedles. This limitation could be overcome by more materials with higher flexibility and mechanical strength [56]. Insertion of microneedles could also be done with the assistance of tools such as a syringe pump [55]. Recently, two-layered and multilayered dissolving microneedles have been fabricated to overcome this limitation [56].

**Figure 3:**
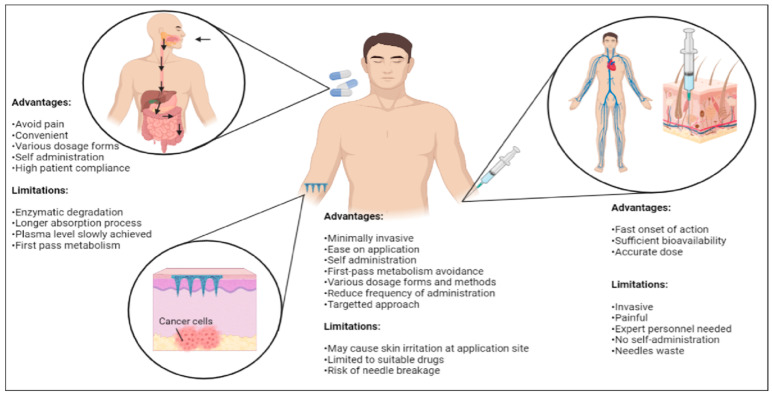
Comparisons among various routes of administrations (oral and hypodermal) commonly involved in conventional drug delivery methods with a transdermal microneedle system for cancer therapy (created using BioRender.com).

Other routes of administration for cancer drug delivery include oral delivery or the use of ointments and topical creams, especially in skin cancer. Oral delivery of cancer drugs (e.g., capecitabine, trifluridine, regorafenib and fluoropyrimidines) are also not suitable and have multiple disadvantages compared to microneedles due to the complex biological environment and internal conditions with pH variability and the presence of enzymes [15,21,57]. Numerous drugs often suffer from poor absorption caused by drug degradation resulting from the first-pass metabolism in the gastrointestinal route and microenvironment changes in pH, food, etc. [15]. Topical creams and ointments such as 5-fluorouracil, imiquimod, resiquimod, ingenol and retinoids also often cause skin irritations. They also show less bioavailability due to the biodegradation in the body and the permeability of the skin, which acts as a barrier towards certain drug molecules [21]. The stratum corneum only shows significant permeability for low molecular weight drugs and lipophilic drugs [21]. Comparatively, microneedles have a faster drug action due to direct release for absorption in systemic circulation [21]. Figure 3 shows the comparisons between microneedles and other conventional drug delivery methods involved in cancer treatments using various routes of administration.

The choice of route of administration is very important for cancer therapy as it determines the desired therapeutic effect, but it is also strongly influenced by various factors such as properties, pharmacokinetics, pharmacodynamics, and effects of the administered substances, as well as the convenience, medical histories of patients and many more [49,58]. Comparatively, microneedles provide a more site-specific approach, circumventing the limitations in current cancer treatments and enhancing the efficiency of cancer drug delivery. Around 75% of drug delivery efficiency has been observed in microneedle-based treatment approaches [49]. Previous studies have also proved that microneedles used in breast cancer therapy allow accurate control of the drug level with spatial and temporal control of drug release avoiding negative effects on other tissues which are usually observed with oral or systemic drug delivery [49].

### 3.2. Self-Administration

The recent COVID-19 pandemic caused delays in several cancer treatments due to the priority setting and allocation of scarce resources, along with inadequate public health workers, especially in second and third world countries. The importance and improvements in personalized medicine have driven the research into self-administered, safe, cost-efficient and convenient drug delivery systems through microneedles. Since microneedles do not require the necessity of the right insertion technique, experienced personnel to perform the procedure, or aseptic materials, the self-administrative approach and ease of administration of microneedles could benefit cancer patients in getting the right treatments at the right time.

Cancer patients would greatly benefit in terms of safety, cost and convenience from the self-administration of microneedles. This is because it could reduce long hours spent at medical facilities waiting for the medical practitioners to administer the drugs and reduces the possibility of any other infections from the hospital. Since microneedles are painless and cause faster healing at the injection site, they can also be easily self-administered compared to a hypodermic needle. Additionally, numerous in vivo investigations, both in animal models and humans, have revealed that microneedle penetration produces negligible bleeding compared to hypodermic needles [18]. This is due to the small size of the micron-scale projections that penetrate the stratum corneum and avoid stimulation of the pain receptors that dwell in the dermis.

The administration of microneedles also reduces the risk of needle-stick injury and cross-contamination, in addition to reducing the burden of disposal [18,21]. An evaluation conducted by the World Health Organization reported that unsafe use and inappropriate reuse of hypodermic needles resulted in approximately 37.6, 39, and 4.4% of hepatitis B, hepatitis C, and HIV cases worldwide, respectively [18]. It has also been reported that more than 100,000 needle stick injuries occur in hospitals each year, which makes microneedles a much better replacement [18]. Microneedles can also be made using biodegradable polymers which allow the self-elimination of any waste left in the human body in the case of a rupture of needles during use, which makes them very beneficial for self-administration.

Insertion of microneedles has proven to result in reduced injury in the injection site and a quicker healing process. In addition, microneedles also allow faster healing of subcutaneous wounds, reduce microbial penetration, prevent edema or erythema, and can be used in combination with pumps for long-term injection [49]. Self-administration of microneedles can also provide cancer patients with the comfort of getting the necessary treatments from being at home. This would greatly aid the elderly in nursing homes, as well as the disabled, as they would not have to travel to and from medical facilities for treatments. In addition, many cancer patients also have concerns about their self-image due to side effects of cancer treatments such as hair loss, weakness, depression and many more, which makes them very resistant to stepping out of their homes.

The self-administrative advantage of microneedles may be limited due to the lower injection accuracy compared to hypodermic needles [49]. This is because the volume of drugs that can be delivered through the intradermal injection can only be around 0.1 mL per injection site and the application cannot be repeated in the same place [18]. Therefore, microneedle administration would not be able to deliver the required volume for high dose drugs. This could be quite challenging in cancer therapies as the dosage or volume of drugs could change according to the physical conditions of patients such as age, sex, and health status such as diabetes, hypertension, and other concomitant health risks [49]. The lack of a fixed drug formulation for patients could hinder the safety and general acceptance of microneedles, especially in cancer therapies, but this limitation can easily be overcome by using different sizes of microneedle patches. Recently, Ripolin et al. [59] successfully demonstrated that larger microneedle patches (16 cm^2^) can be fabricated for medical use [18]. In addition, since the efficiency of microneedles are higher as it locally targets the cancer cells in a shorter duration, a smaller dose would be able to provide the same or better therapeutic approach compared to the hypodermic injections.

Breaking of microneedles in the skin during administration is also feared, but these limitations are very rare and can be overcome with advanced material selection for microneedles [19]. Another major issue would be the misuse of microneedles which could pose a serious issue as it could allow a burst effect in drug release, causing overdose in patients [49]. The drug concentration, a change in microneedle placement angles based on the patient’s position, the effect of external factors such as humidity or cold on the drugs and microneedles, as well as the possibilities of dysfunction of microneedles during self- administration could cause serious problems [49]. This could only be resolved with more research and proper guidelines for patients, caregivers and medical practitioners on the administration of microneedles.

Recent studies are also gearing towards making microneedles suitable for diagnosing cancer and embedding microneedles with biosensors for more personalized and precise cancer therapy. Thus, the self-administration ability of microneedles, along with the advancements in technology, would greatly benefit the patients, family members, medical practitioners and the healthcare industry in the near future.

### 3.3. Painless Treatment Approach

Cancer pain is another serious global concern affecting billions of people worldwide as it is experienced by 64% of patients with an advanced or terminal disease, 59% of patients on cancer treatment, and 33% of patients who had been cured of cancer [60]. Cancer patients also often fear treatments mainly due to the pain and phobia associated with needles (trypanophobia), as well as the side effects caused by the treatments themselves [15,18,21]. There are several pain management strategies in current cancer treatments through pharmacological options in terms of pain-relieving medications available in the market which includes opioids, corticosteroids, anticonvulsants and antidepressants [61,62]. However, limited training, self-management, wrongful understanding of mechanisms of action, pharmacokinetics and inappropriate dosing of the drugs can lead to insufficient pain management and side effects, as well as overdose or addictions, which could even lead to illicit drugs as well as crimes [61,63]. Other noninvasive options are physical care such as massages, exercises, acupuncture, aromatherapy and repositioning, which are safe and may help relieve pain, but have considerably less evidence on the effectiveness towards chronic cancer pain [64], in addition to costing time and money.

Microneedle-based drug delivery offers a minimally invasive approach with less or a tolerable amount of pain, which eventually leads to better patient compliance compared to conventional invasive needle-based drug delivery [21]. Despite the effective systemic delivery of chemotherapeutics achieved by the use of hypodermic needles, the pain associated with hypodermic needle and syringe administration can lead to significant suffering in patients when they have to undergo multiple treatment procedures, especially in pediatric cancer [18]. The perception and acceptability in the pediatric population for immunization through microneedles have also been reported to be positive. This signifies therapy acceptance among children and also parents [21].

Since microneedles only penetrate through the vigorous stratum corneum and the viable epidermis without reaching nerve endings and blood vessels located under the depth of about l mm of this layer [15,21], patients will not be able to feel pain or discomfort during the diagnosis or treatment process. In addition to being painless, microneedles also promote patient compliance through ease of administration. A recent study was done on human subjects to evaluate the skin tolerability and acceptability of administering microneedle patches, and based on the study, 14 out of 15 subjects reported painlessness during administration of the microneedle at the application site, and only one subject rated the pain score and that too was only 1 out of 10 [43]. This strongly proves that microneedle patches are painless.

Microneedles are also being investigated as a means to overcome other biological barriers, releasing drugs in the eye, the oral mucosa, the vaginal mucosa, vascular tissue and so on [21]. This could greatly benefit patients with cervical cancer, ocular melanoma and many more to get painless and efficient treatments at the targeted regions. Painless treatment should be an option for all medical issues, but most importantly in cancer therapy as the patients already have too much on their plate. Thus, taking away the pain of cancer patients could be the biggest gain for them, as well as for the healthcare industry.

### 3.4. Economical

The global burden of cancer continuously grows, exerting tremendous strain on physical and emotional well-being and financial aspects of individuals, families and communities, as well as the health systems [65]. The low- and middle-income countries are especially struggling to manage this burden as compared to the high-income countries that have better survival rates due to accessibility, timely diagnosis, quality of treatment, more public awareness, dietary habits, lifestyle changes, financial security and survivorship care [66].

Although the cost may seem to be one of the major barriers of microneedles in terms of production, administration, transportation and disposal, the benefits of using microneedles which includes reducing multiple dosages of drugs/vaccines, reduction in pain and the number of injections per patient could reduce the overall cost of maintenance. The precision and efficiency of microneedles on drug delivery can also reduce the side effects of drugs and prevent wastage of drugs, in addition to preventing the need for preparation and sterilization for each treatment, which is all more cost-effective compared to the hypodermic needles used in cancer therapy.

Cancer diagnostics and treatments are already considered expensive and the current cancer treatments have specific requirements for the cold chain to carry and store the anticancer drugs, vaccines or other compounds which usually come in multi-dose vials [49]. In addition, certain injectable drugs and vaccines requires dosage adjustment with a dissolvent and the shelf life of dissolved injectable materials are often limited (6 h for vaccines and 8 h for drugs) [49]. The World Health Organisation estimates that half of all vaccines produced globally are wasted, and a large proportion of this wastage is due to failure of the cold chain, especially in developing countries; moreover, previous studies showed that around 60 to 65% of the cost of injecting vaccines and expensive drugs through hypodermic needles can be traced back to the maintenance chain and waste management [49,67]. This makes microneedles a better cost-effective alternative compared to the current hypodermic needles.

Microneedles also have a distinct advantage over liquid vaccines as vaccines can be prepared in a dry state, doing away with the need for refrigeration [36,59]. These dry vaccines are stable at ambient temperatures which would greatly reduce the waste [36,59]. It would be easier to transport and store dry vaccines in microneedles and would also be cheaper to produce [36,59]. This way, microneedles offer an opportunity for the future of vaccination, which is a priority with the rising pandemic situations.

These benefits, when compared, could bring the costs of both microneedles and hypodermic needles to the same level. The use of microneedles and the ease of self-administration could also reduce the need for costly and time-consuming care, the need for medical practitioners, as well as transportation costs, especially for those communities that are far from medical centers, or disabled or elderly patients in nursing care. Moreover, there is highly promising ongoing research to include signaling elements and sensors to detect biological events in microneedles, monitor the physiological conditions of patients and control the drug delivery based on the diagnostic output, which could not only significantly reduce the overall costs, but could also reduce the usual point-of-care costs [49]. The costs of the microneedles are also considered lower compared to hypodermic needles if sustainability is also considered as a point of view. Nevertheless, more information and studies would be needed in terms of the economical aspect of microneedles, especially in the manufacturing process, as a mass production process, the tooling, current micro-machining process capabilities, designation and such should also be deeply considered.

Overall, the advantages of microneedles outweigh hypodermic needles, which makes them very suitable for use in cancer therapy. In addition to hypodermic injections, microneedles are also significantly more beneficial than oral delivery and topical delivery of cancer drugs. We are standing on the cusp of a brighter era with the use of microneedles in cancer therapy, and thus it is highly expected for microneedles to replace hypodermic needles in the near future. However, the limitations should be overcome to reap the full advantages of microneedles for more extensive and efficient use in cancer therapy.

### 3.5. Environmental Sustainability

Medical waste has been a serious issue for decades as it is one of the leading factors towards the increasing carbon footprint and environmental pollution [68,69]. However, it cannot be fully reduced or avoided as most medical devices require proper sterility. The recent COVID-19 pandemic has caused a spike in medical waste with the constant need for masks, gloves and needles due to vaccinations [70]. The unsafe disposal of sharp medical wastes, especially hypodermic needles, is also another major concern [68]. Since there is a need to design more sustainable products, microneedles offer a much more sustainable approach compared to conventional treatment approaches when the concept of sustainability—through its three pillars, which are profit, planet and people—is considered [71].

Most microneedles used in drug delivery are dissolving microneedles and hydrogel-forming microneedles. These types of microneedle arrays could dissolve in the body or degrade easily in the environment, which could eradicate the need for sharp disposal of needles. Polymeric microneedles are also being widely fabricated for drug delivery and other uses, which promises complete biodegradability of the microneedles in the environment as well as low to zero toxicity. Other materials used for the fabrication of solid and hollow microneedle arrays also consist of glass, metal or silicone, which ensures biodegradability and recyclability.

Microneedles also provide a more sustainable approach compared to hypodermic needles as there is a possibility of co-delivery of multiple drugs with microneedles, in addition to reducing the use of various metals and petrochemical compounds used in the production of syringes, intravenous bags and injection tools [69,72]. The environmental impacts of microneedles seem to be reduced, especially during usage and ‘end of life cycle’ stages, but a more definitive study is needed to assess relevant environmental impacts of the selected processes, and more generally of micro-manufacturing despite the common stereotype that the small size induces less material, less production energy and less material to waste. The burden of illness should not be reduced by risking climate change, or we would have to face far worse in the near future. Thus, microneedles seem to be the best fit for a sustainable future in medical care.

## 4. Breaking the Barriers: Microneedles in Cancer Diagnostic, Treatment and Theranostics

Cancer therapy involves various stages which often begin with diagnosis, followed by a variety of treatment options depending on several factors which include type, stages of cancer, affordability, availability of treatments and general health conditions of patients [73]. However, each stage of cancer therapy, right from the diagnosis up to the aftercare, has many barriers in terms of pain, side effects, cost, efficiency and many more, as shown in Table 4 below.

Recently, microneedles have been known to help in overcoming these barriers by reducing the side effects of treatments and relieving pain. The diagnosis of cancer using microneedles has received a lot of attention lately, especially after studies proved that interstitial fluid, particularly of dermal and subcutaneous tissues, has the ability to detect analytes, biomarkers, metabolites and drugs. Diagnosis of cancer through interstitial fluid would avoid repetitive blood extraction and gives more convenience and comfort to cancer patients as it is painlessly accessed through the skin [75]. In addition, the use of microneedles to detect cancer through the interstitial fluid is also simple, fast, safe, low cost and prevents the disposal of sharp needles. Recent studies have also shown that interstitial fluid could detect breast cancer 7 days earlier, and better reflect the progress of early tumors than the conventional blood analysis method [87].

Usually, solid and hollow microneedles would be used to draw the interstitial fluid samples, but there are also studies done with hydrogel microneedles which swell up upon insertion, absorbing the interstitial fluid which was focused on the detection and diagnosis of the presence of both caffeine and glucose [75]. The diagnosis of cancer through an interstitial fluid using a microneedle is still at the initial stages as the extraction techniques are not very feasible for clinical use because blood can be drawn directly from a patient in less than 3 min, while interstitial fluid extraction requires from 10 min up to a number of hours of waiting time and only low sample volumes could be yielded [75]. Therefore, in the near future, it is hoped that this limitation could be overcome so that patients can easily diagnose cancer early without the fear of pain to increase the success rate of treatments and reduce the risk of death often caused by the late diagnosis of cancer.

Treatments for any type of cancer often depend on multiple factors and generally involve either surgery, chemotherapy, radiation, targeted endocrine or molecular therapy and many more [88]. However, recent research has shown that one specific treatment is not sufficient to completely eradicate tumors, prevent the recurrence of cancer cells, and prolong life and symptom palliation [89]. As such, a multimodality approach with a combination of more than one treatment, consisting of systemic therapy that could be preoperative (neoadjuvant), postoperative (adjuvant), or both, is required [88,89]. Despite the advancements in current cancer treatments, a wide range of limitations in terms of physical, emotional, social and psychological wellbeing are still present. All these limitations can be overcome by the localized treatment approach using microneedles, as the current treatments use either oral or intravenous administration where the drugs are most likely to accumulate at different parts of the body before reaching the targeted cancer cells. This would cause more intense side effects and the death of healthy cells, which in turn would necessitate more frequent treatments or higher dosages of drugs, leading to more burdens being carried by the patients.

Microneedles provide highly accurate reproducible results in addition to improved therapeutic advantages. Since microneedles are applied through the skin directly at the targeted area in a minimally invasive manner, cancer patients would feel much more comfortable undergoing multiple rounds of chemotherapy or radiation in a pain-free manner. Moreover, recent studies prove that microneedle patches could be used for combinatory treatments and act as a novel synergetic system, especially in the combination of different treatments or different drugs for a more efficient treatment approach. Table 5 below summarizes the recent studies of microneedles on different treatments.

In addition, cancer immunotherapy, which is considered as one of the first-line treatments, has also been gaining a lot of attention lately. The development of vaccines with enhanced immunogenicity, biocompatibility and safety is considered the Holy Grail in cancer therapy, and such vaccines can be leveraged into personalized medicine [84]. Statistically, the World Health Organization (WHO) also estimates that vaccines can prevent 2–3 million deaths from influenza, meningitis, tetanus and cervical cancer each year [90]. Microneedle-mediated vaccination is considered a potential alternative to traditional vaccination which usually requires hypodermic injection. Moreover, microneedles have been considered particularly promising for the administration of vaccines, taking advantage of the role of the skin as an active immune site, highly rich in antigen-presenting cells, including macrophages, Langerhans cells and dendritic cells, which play a significant role in adaptive immune responses, converting the skin into a favourable place for immunisation [91]. Local immune regulation through microneedles is found to be more effective at lower doses than systemic immune regulation, ensuring it can pass through different in vivo barriers and may also prevent systemic toxicity, overactivation of the systemic immune system and undesirable immune responses caused by the carriers [84]. These macroscale delivery devices have also been explored for the administration of antitumoral gene therapies, antigens, adjuvants, and even antibodies [92].

Although there are still not many attempts of applying microneedles in cancer, it is undeniable that it has obvious advantages and potential in the near future which could break all the barriers of current cancer therapy.

**Table 5:** Summary of recent studies of microneedles on different cancer treatments.

Type of Treatments	Recent Studies
Combinatory treatments	A light-activatable rapidly separable microneedle patch made of polymer containing photosensitive nanomaterials (lanthanum hexaboride) that could quickly deliver doxorubicin (DOX) drug to the skin was used as photo-thermal transducers to repeatedly provide chemotherapy and photothermal therapy to superficial tumors [90].
	Bhatnagar and coworkers developed a polyvinylpyrrolidone/polyvinyl alcohol microneedle patch for the combinatorial delivery of doxorubicin and docetaxel to treat breast cancer tumors where the in vivo studies performed on 4T1 breast tumors bearing mice in this study proved to be more efficient than the single treatment approaches, leading to impaired tumor growth [93].
	Self-assembled nano-dissolving microneedles drug delivery system was successfully constructed for chemo-photothermal combination therapy against melanoma [94].
	Hao et al. [95] combined chemotherapy and photothermal therapy for the development of a PEGylated gold nanorod coated poly(l-lactide) microneedle system in order to enhance the antitumor efficiency of docetaxel-loaded MPEG-PDLLA micelles for the treatment of A431 tumors [95,96].
Immunotherapy	Encapsulation of DNA vaccines within microneedles as delivery vehicles protected the vaccines from the hostile environment in vivo, including omnipresent nucleases, increasing the half-life of vaccines while generating long-lasting immune stimulatory effects [84].
	Microneedle administration of antibodies showed that the concentration of antibodies was 2 times higher than that of the control, and the T cells were more responsive to HPV-16 oncogenic antigen expressing cells (TC-1) (IFN-γ levels in control ≈ 250 pg/mL and ≈530 pg/mL. This enhanced immune response prevented the establishment of cervical tumors in 4 of the 9 mice treated with microneedles [92].
Gene therapy	A polyvinylpyrrolidone microneedle patch loaded with E6/E7 pDNA RALA nanoparticles for the gene therapy of cervical cancer was developed by Ali et al. [97].
	A hyaluronic acid-based microneedle array for mediating the delivery of anti-PD1 antibody (aPD1), and -methyl-DL-tryptophan (1-MT) to B16F10 melanoma tumors was also produced, in which the aPD1 targets the PD-1 receptors expressed by T cells, and therefore avoids the cancer cells inhibitory signaling that prevent the T cells activation [98].

## 5. Future Direction of Microneedles in Cancer Care

The scope for the future direction of microneedles should first be in prioritizing the safety and performance with more preclinical and clinical investigations, especially since cancer is a chronic illness and the use of microneedles in cancer therapies is still a novel idea, and the study on microneedle capabilities in the human setting is considered instrumental. The parameters of microneedles such as mechanical strength, materials used in fabrication and immunogenic reactions should be mainly considered, while also improving microneedle development to scale-up microneedles from the laboratory to the market for commercialization in cancer therapies. In addition, there are several challenges, especially in terms of cost, regulatory aspects such as pharmacopeial standards and good manufacturing practice (GMP) guidelines, quality control and the possible requisite for a sterile manufacturing process, as well as regulatory guidelines regarding patient use, packaging, waste, ease of application and additional safety for microneedles, which should be well studied and highlighted in the future studies due to the novel nature of microneedles [18,99]. Patients as well as medical practitioners might also need a lot of training and practice on techniques for self-administration of microneedles to avoid unnecessary complications.

The future direction of microneedles in cancer therapy is also moving towards a more personalized and precision-based approach as it would offer more customized care for patients with minimal pain and supervision. This would be a great approach in the near future as we would have more advanced technology. Theranostics have emerged in recent years as a method of increasing the personalization of therapies by combining diagnostic and therapeutic capabilities into a single system. This field is growing and holds the promise of allowing minimally invasive disease monitoring, ultimately integrating closed-loop devices in which detection and therapy are achieved in a minimally invasive way. In particular, microneedles, with their emphasis on a painless and local targeting approach to tumors through dermal layers, have offered a convenient avenue for theranostic development.

**Figure 4:**
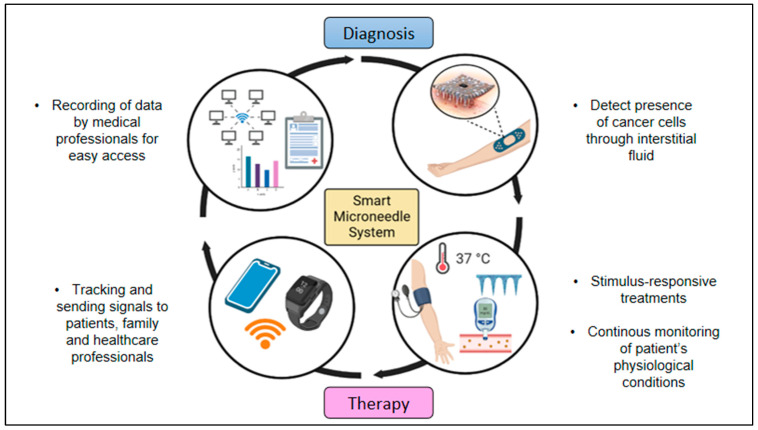
Schematic illustration of smart microneedle system that consists of a loop system from diagnosis to cancer therapy (created using BioRender.com).

Researchers are also constantly trying to design and construct smart microneedle systems, such as a microneedle patch that is embedded with biosensors and has wearable applications or tattoo-based application. This system would be able to offer a multifunctional approach that would bring cancer therapy to a whole new level. This could also allow the transmission of personalized health data wirelessly, as well as achieving personalized diagnostics through telemedicine and cloud medicine [15]. The biomaterials in a smart microneedle system can be specifically designed to be sensitive to a specific stimulus present in the tumor microenvironment, e.g., temperature, pH, the wavelength or intensity of incident light or an electrical or magnetic field; and to then respond in active ways including changing their structure for drug delivery, radioprotection, priming an immune response, or other functions that have the potential to enhance cancer diagnosis and therapy [100]. The delivery of drugs through the microneedles could also be monitored, and this would greatly aid family members and medical practitioners to keep track of the conditions of the patients. This system could also be adapted as a blockchain system in future medical care to allow easy transmission of health data with traceability and trackability features which could also maintain more transparency and avoid any problems that may arise. Figure 4 shows the schematic diagram of a smart microneedle system which would greatly benefit future cancer therapy.

In addition, this system would enable microneedles to be used as a wearable platform for molecular detection or monitoring as they have been integrated with biosensors capable of detecting metabolites, electrolytes and other clinically relevant targets [75]. This could prevent the frequent collection of blood/interstitial fluid samples for the quantification of glucose or viral antigens, respectively [91]. An elegant approach using polymeric hydrogel-forming microneedles that could soak the interstitial fluid could be easily used for diagnosis in a painless and hassle-free manner [91].

The data collected through the biosensors could be designed to be transmitted to smartphones. Sophisticated features such as in-built high-quality camera lenses, wireless connectivity, mobile applications that aid productivity, and video streaming would enable a very connected therapeutic approach [101]. This system is still a novel idea for microneedle patches, but the concept of using biosensors for medical treatments and connecting it with smartphones has been previously tested. A sweat diagnostics system for cystic fibrosis using a low-cost smartphone-based chloridometer equipped with a novel citrate-derived fluorescence sensor was developed [102,103]. The sensor material demonstrated a wide linear range of 0.8–200 mM chloride and a diffusion-limited response time [102,103]. Analytical and clinical validation was performed with sweat from individuals with and without cystic fibrosis, demonstrating convenient sweat diagnostics with reliable detection of cystic fibrosis [101]. However, only the continuous glucose monitoring systems (CGM’s), such as the Dexcom, Freestyle Libre and the Minimed, are, to the best of the authors’ knowledge, the only wearable devices on the market capable of monitoring at a molecular level [75]. More priority and in-depth studies on microneedles and their application to cancer would provide painless gains and without doubt aid in reducing the global cancer mortality rate.

## 6. Conclusions

The application of microneedles in cancer therapy has opened hopes of providing a pain-free treatment approach for patients in the near future. Several aspects of the microneedle-based delivery approach for cancer treatments as discussed above have been uncovered, which could pave the way for novel therapeutic modalities. Precision and personalized medicine which is customized based on patients’ history and genes is making significant headway and there may be a lot of advancement to be included in the designing and construction of microneedles to make it more outstanding in the near future. However, the existence of inherent limitations in the area of transdermal microneedle-based delivery should be strongly considered, and further attempts in understanding the mechanisms associated with efficient delivery are needed. Many significant advances in the research, diagnosis and treatment of cancer have been made in recent years despite the challenges, and are expected to improve further in upcoming years, especially with the breakthroughs in computer science, artificial intelligence, virtual reality and the internet of things (IoT). Since the ultimate goal of cancer therapy is to maximize damage to the cancer cells, while minimizing the negative and undesirable effects, we are already standing on the cusp of a brighter era with the use of microneedles in cancer therapy.

## Figures and Tables

**Table 1:** Common examples of commercially available various types of microneedle patches for various applications.

Brand Name and Manufacturer	Types of Microneedles	Applications	References
DrugMAT and VatMAT; TheraJect Inc., Fremont, CA, USA	Dissolving microneedles	Transdermal patches for large molecules more than 500D (proteins, vaccines and genetic materials)	[22,23]
Nanoject^®^; Debiotech, Lausanne, Switzerland	Dissolvable peptide microneedle patch	Intradermal and hypodermic drug delivery and for interstitial fluid diagnostics	[20]
Macroflux; Zosano Pharma Inc., Fremont, CA, USA	Metal microneedle patch	Intracutaneous microneedle system for peptide and vaccines Rapid hydrophilic drug-coated patches Microneedle size ~ 200–350 µm	[24]
Onvax; Becton Dickinson, Franklin Lakes, NJ, USA	Microneedle array patch	Plastic micro-projections Microneedle size ~ 200 µm	[25]
MicroCor^®^ PTH(1–34) Corium International Inc., Boston, MA, USA	Dissolving microneedles	Made from unique polymer combinations and compositions Contains active ingredient for the treatment of osteoporosis	[23,26]

**Table 2:** Advantages and limitations of different types of microneedles (solid, hollow, coated and dissolving) for use in cancer therapy (modified from [27,28]).

Type of Microneedle	Advantages	Limitations
Solid	Mechanical strength Physical stability Reasonable drug loading	Poor dose accuracy Requirements of rapid healing after application Potential for infections due to reuse Poor biocompatibility
Coated	Mechanical strength	Peeling during insertion Poor biocompatibility Dose limitation Formulation migration during manufacturing and storage
Dissolving	Low cost manufacturing Ease of manufacturing Controlled drug release profile One-step application	Poor mechanical strength Physical stability and biocompatibility Dose limitation
Hollow	Dose accuracy Reasonable drug loading Constant flow rate	Clogging Ingressing body fluids Requirement of prefilled syringe Poor mechanical strength Potential for infection due to reuse Poor biocompatibility
Hydrogel forming	No residual excipients in the skin after removal Easy to manufacture Reasonable drug loading Controlled drug release profile	Poor mechanical strength and physical stability Ingressing body fluids

**Table 3:** Summary of advantages and limitations of materials and fabrication methods used for different types of microneedles (solid, hollow, coated and dissolving).

Materials	Fabrication Method	Types of Microneedles	Advantage	Disadvantage	References
Metal	Wet etching, lithography, metal injection molding, laser machining	Solid, Hollow	High mechanical strength, biocompatible	May induce allergic reactions, produce sharp waste	[28,31,32,33]
Bio-ceramic	Micro-molding	Solid	Resist towards chemical	Low tensile strength, may cause irritation if breaks inside the skin	[34,35,36]
Silicon	Deep reactive ion etching	Hollow	Microneedle of various shapes and sizes can be produced as it is flexible	Brittle, fabrication process is time-consuming, high cost	[19,37]
Sucrose, Hyaluronic acid	Micro-molding, droplet-born air-blowing	Dissolving	Biodegradable, low toxicity, able to stabilize protein molecules, good mechanical strength	Low mechanical strength	[38,39,40,41,42]
Polylactic acid (PLA), Poly-L-lacticacid (PLLA), Polyvinyl alcohol (PVA), Polyvinylpyrrolidone (PVP), Polycaprolactone (PCL)	Thermal micro-molding, micro-molding, centrifugal lithography	Solid, Coated, Dissolving	Biodegradable, inexpensive	Low mechanical strength	[32,35,38,43,44,45,46,47,48]

**Table 4:** Barriers in different stages involved in cancer therapy.

Stages in Cancer Therapy	Description	Barriers	References
Diagnosis	Medical history, local examination of tumor area and repeated analysis of bio-fluids (e.g., blood, urine and saliva) through lumbar punctures, bone marrow aspirations, biopsy and venepunctures	Frequent painful procedures Lack of awareness and knowledge Depression Anxiety Fear Financial burden Preference for alternate treatments Dependency/burden to family Loss of job Loss of quality of life	[74,75,76]
Surgery	Removal of tumor, usually performed as the only treatment prior to or after chemotherapy.	Loss of mobility Pain Hematoma Infections Loss of quality of life	[77]
Chemotherapy		Mucositis, Gastritis, infection Peripheral neuropathy Hair loss Fatigue Nausea, Constipation, Death of healthy cells Drug resistance	[76,78,79,80,81]
Radiation	High-powered energy beams, such as protons, electrical energy or X-rays, target and destroy cancer cells	Severe damage to normal cells, Skin reactions (e.g., dermatitis, burns, damage to nerve fibers, myelopathy, plexopathy and numbness)	[73,75,82]
Photothermal	Employing plasmonic nanoparticles localized in tumors as exogenous energy absorbers that convert laser energy into heat, causing irreversible cellular damage and subsequent tumor destruction	Nanoparticles rapidly cleared from the tumor sites Accumulated in the liver, spleen and other organs after irradiation Requires reinjection to achieve an adequate concentration within the tumors	[83]
Immunotherapy	First-line treatment that strengthens patient’s own immune system to naturally fight, defend and kill cancer cells Immune checkpoint blockade and cancer vaccines	Weak immune response Possibility of undetected contaminations Need for cold chain during storage and transit of vaccines	[84,85]
Targeted		Heart impairment Diarrhea Shortness of breath	[77,86]

## Data Availability

The data presented in this study is openly available.
